# Validating the role of PTGIS gene in colorectal cancer by bioinformatics analysis and in vitro experiments

**DOI:** 10.1038/s41598-023-43289-2

**Published:** 2023-10-01

**Authors:** Hui Ding, Kai-yun Wang, Si-yang Chen, Kai-Wen Guo, Wen-hong Qiu

**Affiliations:** 1 Medical College of Wuhan Science and Technology University, Wuhan, 430065 People’s Republic of China; 2 Department of Immunology, School of Medicine, Jianghan University, Wuhan, 430056 People’s Republic of China

**Keywords:** Cancer, Immunology

## Abstract

Prostaglandin I2 synthase (PTGIS) is a member of the cytochrome P450 family. Studies have revealed that differential expression of the PTGIS gene is closely related to the pathological and physiological processes of many diseases, including breast cancer, oral squamous cell carcinoma, and head and neck cancer. However, the mechanism of action of the PTGIS gene in colorectal cancer is not fully understood. This study explored the role of PTGIS in colorectal cancer through comprehensive bioinformatics analysis and in vitro experiments, and found that the expression of PTGIS gene in colorectal cancer tissue was significantly lower than that in normal colorectal tissue (P < 0.05), and high expression of PTGIS gene was associated with poor prognosis in patients (P < 0.05). The KEGG results showed that PTGIS-related genes were mainly enriched in metabolic pathways, arachidonic acid metabolism, steroid biosynthesis, and cancer pathways. The expression of PTGIS may be related to immune infiltration. Cell experiments showed that PTGIS was expressed at a lower level in cancer. Overexpression of PTGIS inhibited apoptosis and promoted proliferation, invasion, and migration ability of SW480 colorectal cancer cells. Analysis of the PTGIS gene in this study provides a theoretical basis for further exploring the pathogenesis of colorectal cancer and finding more accurate new targets for early screening and treatment of the cancer.

## Introduction

The PTGIS (prostaglandin I2 synthase) gene is a member of the cytochrome p450 superfamily (CYP450) gene, family 8, subfamily A, polypeptide 1, PTGIS is also known as CYP8A1, prostacyclin synthase, PGIS, CYP8, PTGI, is a gene encoding protein, located on chromosome 20q13.11-q13.13, the total length of about 60 kb, consisting of ten exons^1^. The protein encoded by this gene is prostaglandin I2 synthase, a membrane protein found in the endoplasmic reticulum.The enzyme is a monooxygenase that catalyzes drug metabolism, steroids, cholesterol and other lipid synthesis. Studies have shown that differences in PTGIS gene expression are closely related to the pathophysiological processes of various cancers, including breast cancer^2^, ovary cancer^3^,kidney cancer^4^, bladder cancer^5,6^, squamous cell carcinoma of the lung^7^, etc.

According to the latest global cancer statistics compiled by the International Cancer Agency, In 2020 there are approximately 19.3 million new cancer cases worldwide and nearly 10 million deaths from cancer^8^. Due to the aging of the world population, social and economic development and other factors, global cancer incidence and mortality show an increasing trend. Malignant tumors have now become one of the main causes of death in all regions of the world^9^. However, the mechanism of the PTGIS gene on cancer is not very clear. This paper intends to provide theoretical support for early screening, early treatment and prevention of some cancers by combining bioinformatic analysis with cell experiments.

## Materials and methods

### Pan-oncogene expression and prognosis

To study PTGIS expression in pancancer, data was first obtained from TCGA and GTEx databases using GEPIA 2 (http://gepia2.cancerpku.cn). By analyzing TCCG and GTEx data, it can quickly provide customizable functionality. Also, filter the PTGIS gene entry in the Oncomine Oncogene Gene Chip Database (https://www.oncomine.org/) and the Integrated Data Mining Platform, which is currently the largest oncogene chip database available. It can be used to analyze differences in gene expression and is useful for exploring biomarkers and new therapeutic targets. The GEPIA 2 Survival Map module was used to obtain the meaning of overall survival (OS) from PTGIS in 33 tumor types.

### Genetic alteration

In the cBioPortal web (https://www.cbioportal.org/), the change frequency, mutation type and copy number change (CNA) of PTGIS across all TCGA tumors were found in the Cancer Types Summarymodule.

### Differential expression and prognosis in colorectal cancer

To further verify the differential expression of PTGIS genes in colorectal cancer tissues and normal colorectal tissues, we performed differential expression analysis of PTGIS in another commonly used oncogene chip database—UALCAN (http://www.ualcan.path.uab.edu/). GEPIA 2 was again used to analyze the difference in PTGIS expression between colorectal cancer and normal tissue. We performed HPA (https://www.proteinatlas.org/) to compare the protein expression of PTGIS between normal colorectal tissue and colorectal cancer tissue. The LinkedOmics database (http://www.linkedOmics.org/) is a multi-omics database of global proteomics data, commonly used to aggregate intra-tumor and inter-tumor multi-omics data compare.

### Cell culture

Human colorectal cancer cell SW480 cells were preserved and given to us by the Life and Science Health Institute of Wuhan University of Science and Technology. Human colorectal cancer cell line HCT8 were purchased from China Typical Culture Preservation Center (Wuhan, China). Human normal colorectal epithelial tissue cell line FHC was purchased from Shenzhen Huatuo Biotechnology Co., Ltd. SW480, HCT8 and FHC cells were cultured in RPMI 1640 complete culture medium (containing 10% fetal bovine serum and 1% double antibody) and then cultured in a cell incubator adjusted to 37 °C and 5% CO_2_.

### Lentiviral infection

Firstly, we constructed the plasmid PLVX-PTGIS-EF1-PURO. The designed PTGIS primers: Forward: 5-GATCTATTTCCGGTGAATTCGCCACCATGGCTTGGGCCCGCG-3; Reverse: 5- GCGGCCGCTCTAGAACTAGTTCATGGGCGGATGCGGTAG-3. After annealing, the primers were ligated to the linearized vector, which was digested with T4 ligase by the plasmid PLVX-EF1-EGFP-PURO. The plasmid was transfected into 293 T cells and the supernatant was collected. SW480 cells were infected with the supernatant and polybrene lentivirus, and screened with puromycin.

### Quantitative real‐time polymerase chain reaction (RT-qPCR)

Total RNA was extracted from cells by TRIzol. Reverse transcription was performed according to the manufacturer's instructions. Real-time PCR was performed using SYBR GreenER qPCR SuperMix Universal and the PCR system. Real-time PCR (qPCR) primer sequences: GAPDH: Forward: 5-CCAGCAAGAGCACAAGAGGAAGAG-3; Backward: 5-GGTCTACATGGCAACTGTGAGGAG-3.PTGIS: Forward: 5-ACTGTTGCTGCTGCTGCTACTG-3; Back: 5-GAGGAAGATGGCATAGGCATGGAAG-3.

### Western blot (WB)

FHC, SW480 and HCT8 cells were collected and lysed with RIPA lysate to extract total protein. The total protein concentration was determined by the BCA kit. 20 μg protein was removed for electrophoresis and PVDF membrane transfer. The protein was sealed with 5% skim milk powder blocking solution for 1 h, then the corresponding primary antibody (PTGIS and actin, at a dilution ratio of 1: 1000) was added and incubated at 4 °C for 14 h. Wash three times with TBST, 5 min each time. Then added HRP-labeled secondary antibody (goat anti-rabbit IgG), incubated for 1 h at room temperature. Washed three more times with TBST. Ultra-High Sensitivity ECL kit was used for color rendering, a gel imager was used to observe the bands and take pictures, and Image J software was used to analyze the relative expression levels of each protein group.

### MTT assay for cell proliferation

Placed the cells in a 96-well plate (200 µl/well) with complete medium equivalent to 5000 cells/well and each group had 6 sub-wells. Continue cultured for 3 days in a 37 °C and 5% CO_2_ incubator. The activity of the cells in each group was detected by the MTT method on days 1, 2 and 3 after planking. The individual steps were as follows: added 20 µl MTT/well (MTT storage solution: 5 mg/ml) to the 96-well plate, incubated in the incubator for 4 h, removed the supernatant, added 150 µl DMSO, shaked on the shaking table for 10 min (80 rpm) and then used the enzyme label to determine the absorbance value of each sample at OD490nm. The proliferative activity of the cells in each group was recorded and analyzed.

### EdU detects cell proliferation

Placed the cells in a 6-well plate (2 ml/well) with complete medium. Continue cultured for 2 days in a 37 °C and 5% CO_2_ incubator. The activity of the cells in each group was detected by the fluorescence microscope on days 2 after planking. The individual steps were as follows: added 2 ml EdU/well (EdU storage solution: 20 µM) to the 6-well plate, incubated in the incubator for 2 h, removed the supernatant, added 1 ml paraformaldehyde to fix for 15 min, shaking washed on a shaker three times for 5 min(80 rpm) esch. Removed the supernatant, added 1 ml 0.3%Triton, incubated for 15 min. After washing three times, added 0.5 ml Click Additive Solution, and incubated at room temperature in the dark for 30 min. Add 1 ml Hoechst, incubated in the dark for 30 min. Subsequently, the cell proliferative ability was detected by fluorescence.

### Flow cytometry apoptosis detection

Cultured the cells until their confluency reached 80%, and aspirated the supernatant medium. Rinsed cells with PBS, digested with trypsin, centrifuged for 5 min at 1200 rpm and collected cells. Then centrifuged the previous supernatant medium together with the cells obtained by the above centrifugation. Resuspend the cells in PBS and repeated twice. Prepared the staining solution according to the standard of 300 µl of staining buffer (Solution A) and 3 µl of staining solution (Solution B) from each well, transfered the prepared mixture to the flow-through tube, avoid light for 30 min, performed a flow-based Detection and analyzed the data.

### Transwell migration assay

Cell migration was assayed with a transwell chamber device, the lower chamber was filled with 600 µl of RPMI-1640 containing 20% FBS. A total of 1105 cells in 200 µl of serum-free RPMI-1640 were seeded in the upper well and each incubated with Tagitinin C for 24 h at 37 °C. Migrated cells were fixed with 4% paraformaldehyde and stained with 1% crystal violet. Pictures were taken using a microscope and the migrated cells were counted.

### Wound healing assay

The SW480 cells were cultured in six-well dishes. Scratched the cell monolayer was with a pipette tip when the cell density reached more than 90%. Then washed the dishes twice with PBS and incubated with medium containing 10% fetal bovine serum. All cell lines were cultured in a humidified incubator at 37 °C and 5% CO_2_ and migrated for 24 h and 48 h.

### Gene pathway correlation

RNAseq data and corresponding clinical information on colorectal tumors were obtained from the Cancer Genome Atlas (TCGA) database. The genes involved in the respective signaling pathways were collected and analyzed using the GSVA package of the R software. The parameter method = ssgsea was selected. Finally, the correlation between genes and pathway scores was analyzed by Spearman’s correlation.

### Immune infiltration

RNAseq data and corresponding clinical information for colorectal tumors were obtained from the Cancer Genome Atlas (TCGA) database. In order to obtain reliable immunocorrelation assessment, we used immunedeconv, an R package that integrates six latest algorithms, including TIMER, xCell, MCP-Counter, CIBERSORT, EPIC, and quantiseq. SIGLEC15, IDO1, CD274, HAVCR2, PDCD1, CTLA4, LAG3, and PDCD1LG2 are immune checkpoint-related transcripts. We extracted the expression levels of these 8 genes to monitor the expression of immune checkpoint-related genes.

### String database (https://cn.string-db.org/)

Used the string interaction network to draw the associated protein network diagram of PTGIS genes. Then used KEGG enrichment to view the key biological processes they are involved in.

### Statistical analysis

Statistical analyzes were performed with the GraphPad Prism 9 software. Data were expressed as mean standard deviation (SD) and analyzed by one-way ANOVA or Student's t-test. Differences with P values < 0.05 were considered statistically significant.

## Results

### Expression and prognostic pattern of PTGIS in the pan-cancer perspective

**Figure 1:**
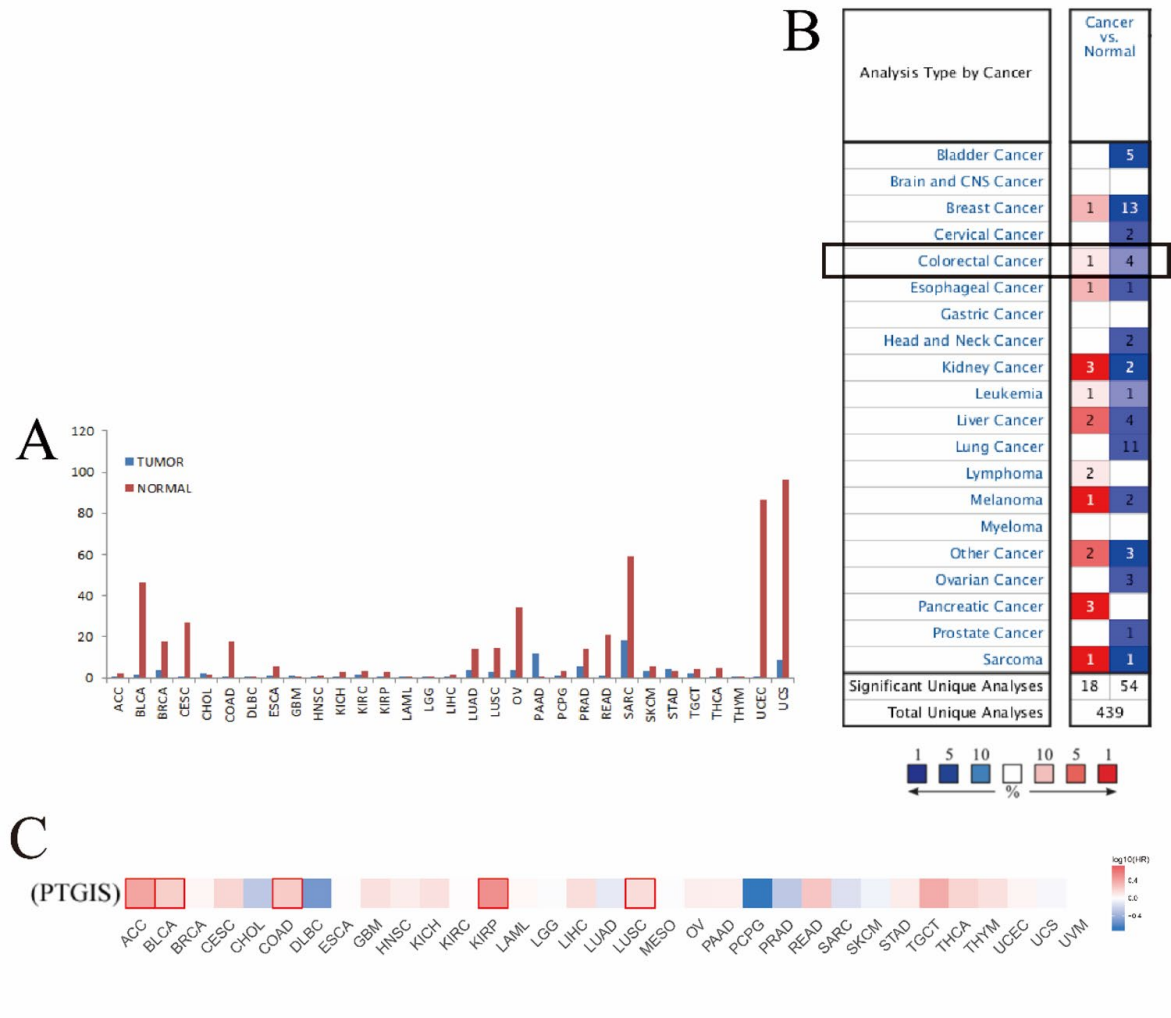
Expression and prognostic pattern of PTGIS in the pan-cancer perspective. (**A**) The expression of PTGIS from the perspective of pan-cancer; (**B**) in Oncomine database, the expression of PTGIS gene in tumor tissue is shown. The box shows the expression of PTGIS gene in colorectal cancer; (**C**) the prognosis of PTGIS from the perspective of pan-cancer.

The gene expression profile across all tumor samples and paired normal tissues were shown in (Fig. 1A), PTGIS was significantly upregulated in 1 out of all 33 cancer types compared with normal tissue, and it was significantly downregulated in 17 out of all 33 cancer types. These data showed that PTGIS mRNA expression was abnormally expressed in various cancers. The differential expression of PTGIS gene in different cancer types and normal tissues was analyzed in Oncomine database. The results showed that the expression of PTGIS gene was up-regulated in red and down-regulated in blue. The darker the color, the higher the meaning. The number on the graph represents the number of data sets with statistical significance, and PTGIS was low in colorectal cancer (Fig. 1B). According to the expression of PTGIS, the TCGA data set was divided into a hige-expression group and a low-expression group. Red indicates that the higher the expression of PTGIS, the worse the prognosis, while blue indicates the opposite. Results showed that in ACC, BLCA, COAD, KIRP, and LUSC, high expression of PTGIS was associated with poor prognosis, specifically poor overall survival (Fig. 1C).

### Genetic alteration analysis

**Figure 2:**
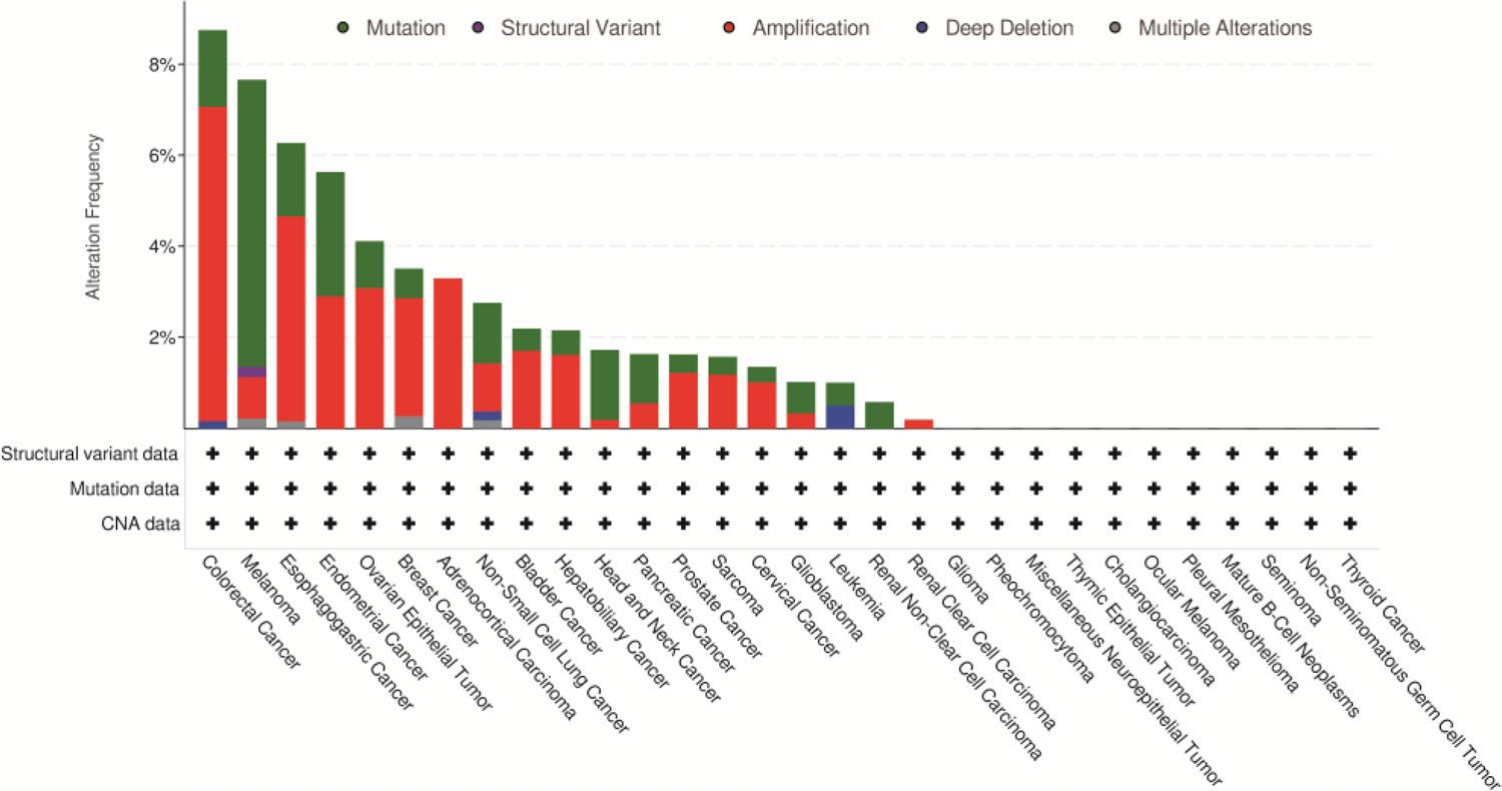
Mutation analysis of PTGIS from the perspective of pan-cancer.

The frequency of genetic variation of PTGIS gene in the list of tumor types was analyzed in different tumor samples, The most frequent change of PTGIS (> 8%) occurred in patients with colorectal cancer, and “amplification” was the primary type of alteration (Fig. 2).

### Differential expression and prognosis in colorectal cancer

**Figure 3:**
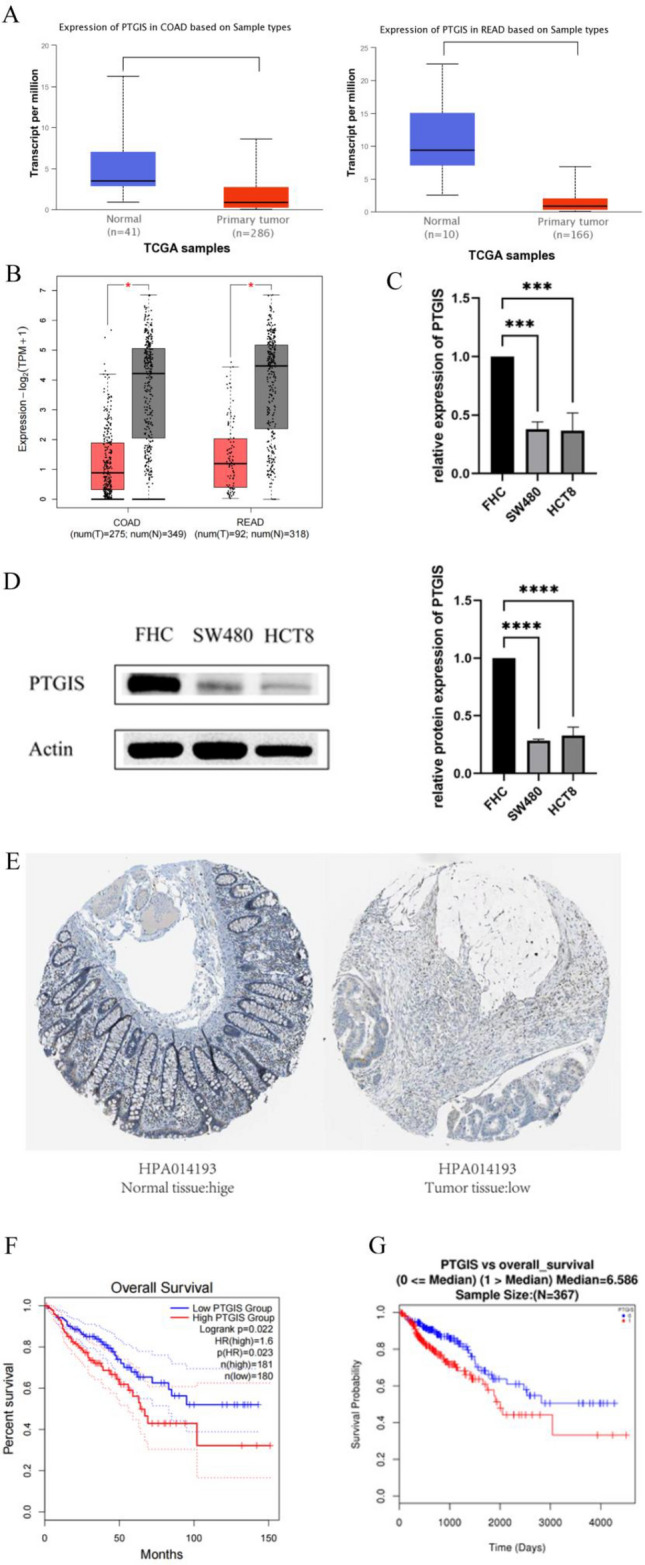
(**A**) The expression of PTGIS in colon adenocarcinoma (COAD), rectal adenocarcinoma (read) and normal tissues in Ualcan database; (**B**) the expression of PTGIS GEPIA 2 database of colon adenocarcinoma (COAD), rectal adenocarcinoma (read) and normal tissues; (**C**) q-PCR was used to detect the mRNA expression of PTGIS in normal colorectal cells FHC, colorectal cancer cells SW480 and HCT8; (**D**) WB was used to detect the protein expression of PTGIS in normal colorectal cells FHC, colorectal cancer cells SW480 and HCT8; (**E**) the protein levels of PTGIS based on Human Protein Atlas between Normal tissue and tumor tissue; (**F**) the relationship between PTGIS gene expression and prognosis in GEPIA 2 database; (**G**) objective to investigate the relationship between PTGIS gene expression and prognosis in LinkedOmics database.

In the UALCAN database, PTGIS gene expression was lower in colon adenocarcinoma tissues (N = 286) compared with normal colorectal tissues (N = 41), and the difference was statistically significant (p = 1.900700e−03). Comparing normal rectal tissues (n = 10) and rectal adenocarcinoma tissues (N = 166), PTGIS gene was found to be significantly lower in rectal adenocarcinoma (p = 3.8442999999991e−06). (As shown in Fig. 3A). There are a total of 1034 associated samples in the GEPIA 2 database, the red box was the normal group, and the gray box was the tumor group including a comparison of colon adenocarcinoma tissue (n = 275) with normal tissue (n = 349) and a comparison of rectal adenocarcinoma tissue (n = 92) with normal tissue (N = 318) respectively. Obviously, compared with normal tissues, the PTGIS gene showed low expression in colon adenocarcinoma and rectal adenocarcinoma, and the difference was statistically significant (P < 0.05) (Fig. 3B). This was valitated by the results of the q-PCR. Compared with normal colorectal cells (FHC), the expression of PTGIS mRNA was lower in colorectal cancer cells (HCT8, SW480) (Fig. 3C). This was shown again by the results of Western blot (Fig. 3D) (Supplementary Information). Immunohistochemical staining of HPA also revealed that PTGIS protein was downregulated in colon tissue (Fig. 3E). There are a total of 361 samples analyzing the effects of PTGIS gene expression in colorectal cancer patients in the GEPIA2 database, including 181 high-expression samples and 180 low-expression samples. It was evident that the overall survival rate of patients with high expression of the PTGIS gene is lower than that of patients with low expression (P < 0.05) (Fig. 3F). In the LinkedOmics database, the overall survival rate of patients with low PTGIS gene expression appeared to be higher than that of patients with high expression (P < 0.05), and the difference was statistically significant (Fig. 3G).

### The effect of overexpression of PTGIS on colorectal cancer proliferation and apoptosis

**Figure 4:**
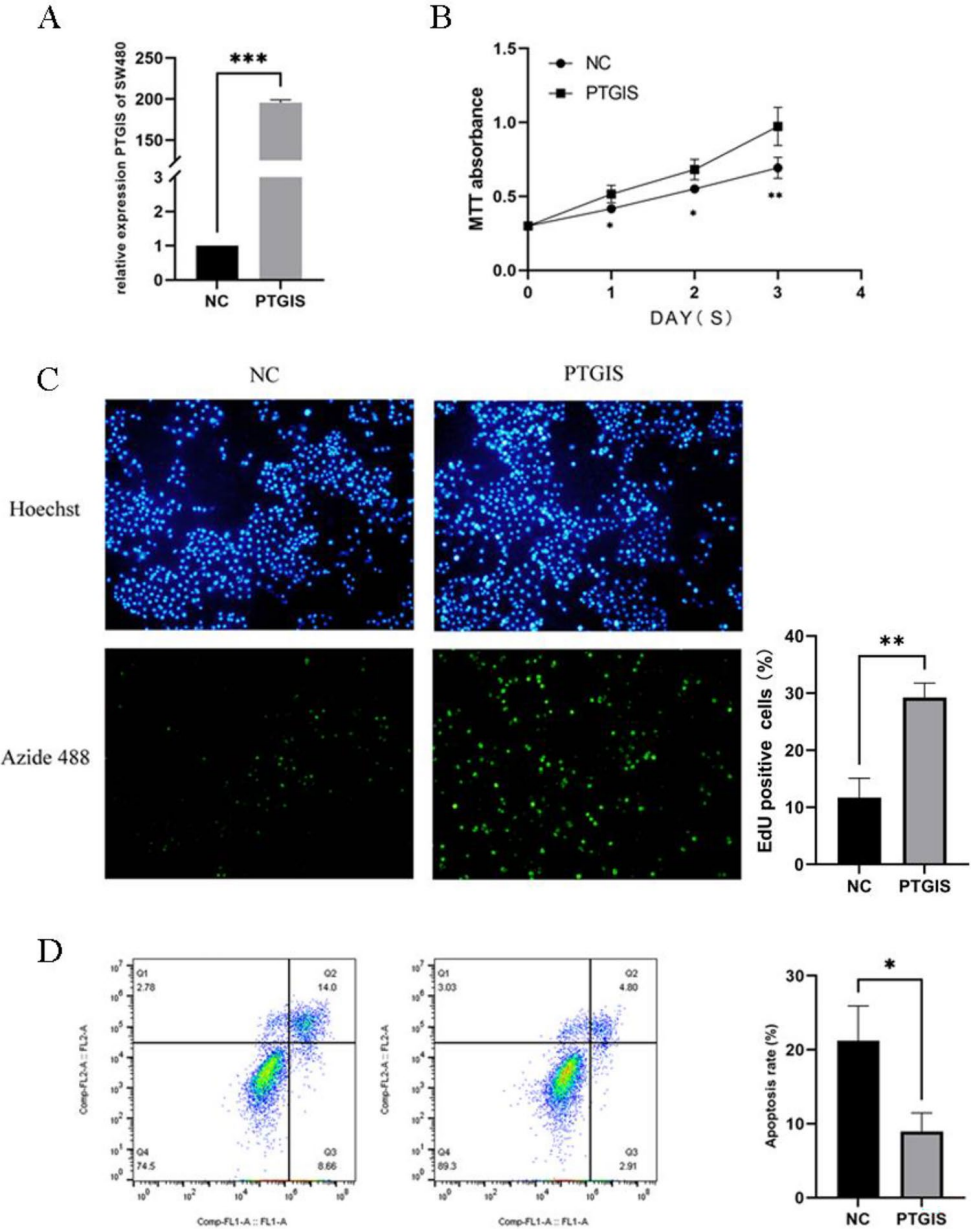
The effect of overexpression of PTGIS on colorectal cancer proliferation and apoptosis. (**A**) To detect the overexpression effect of PTGIS in SW480 cells by q-PCR; (**B**) the effect of PTGIS overexpression on the proliferation of SW480 cells by MTT assay; (**C**) the proliferation activity of single SW480 cell was detected by EdU assay; (**D**) the effect of PTGIS overexpression on apoptosis of SW480 cells by flow cytometry.

The results showed that the mRNA expression level of the experimental group (PTGIS) was significantly upregulated, proving that the SW480 overexpression cells were successfully constructed (Fig. 4A); compared with the control group (NC), the experimental group had stronger cell proliferation ability, higher activity (Fig. 4B); the results showed that there were more EdU + SW480 cells in the PTGIS group, and the proliferative ability of single cells in the PTGIS group was stronger than that in the control group(NC) (Fig. 4C), and weaker apoptosis (Fig. 4D).

### Effect of PTGIS overexpression on invasion and migration of colorectal cancer

**Figure 5:**
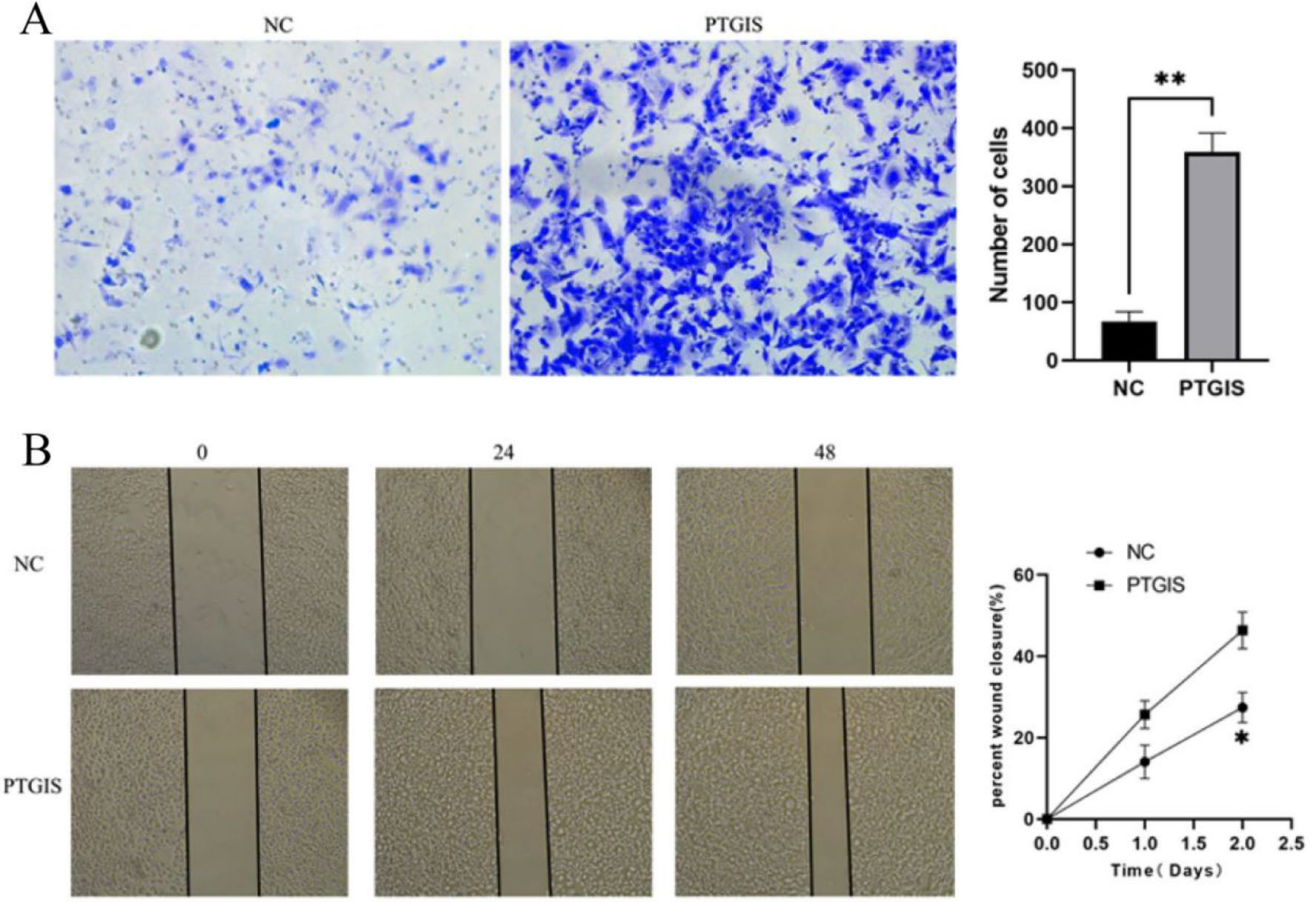
Effect of PTGIS overexpression on invasion and migration of colorectal cancer. (**A**) The effect of PTGIS overexpression on SW480 cell invasion; (**B**) the effect of PTGIS overexpression on SW480 cell migration.

The results showed that compared with the control group (NC), the experimental group (PTGIS) had stronger cell invasion ability (Fig. 5A) and migration ability (Fig. 5B) and increased the cell apoptosis.

### Spearman correlation analysis between PTGIS gene and EMT pathway score

**Figure 6:**
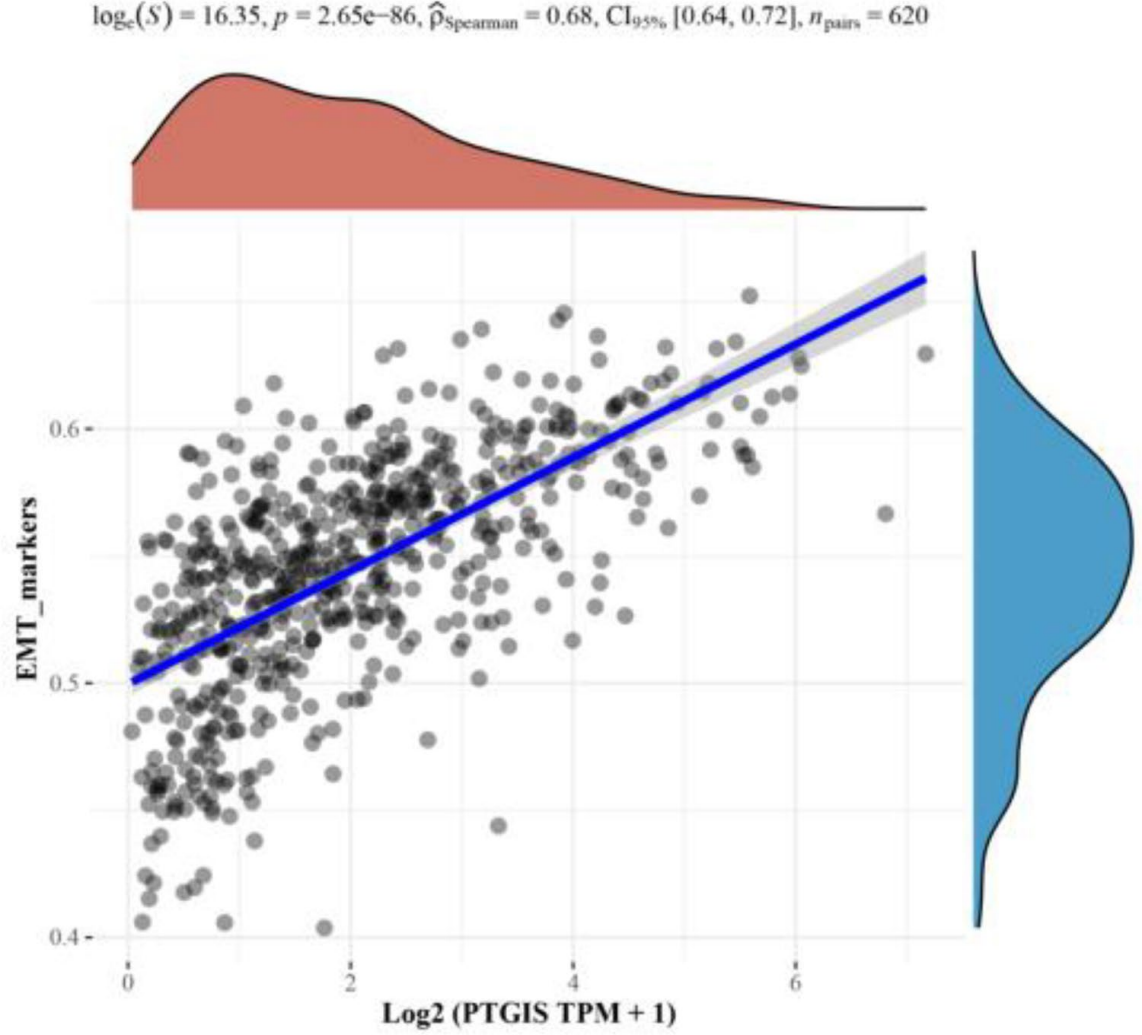
Spearman correlation analysis between PTGIS gene and EMT pathway. X axis: represents the expression of genes, the Y axis: represents the pathway score.

The X axis represents the expression of genes, the Y axis represents the pathway score, and the density curve on the right represents the distribution trend of the pathway score. The upper side density curve is the distribution trend of gene expression. The results showed that the expression of PTGIS gene was positively correlated with EMT (Fig. 6).

### Immune infiltration

**Figure 7:**
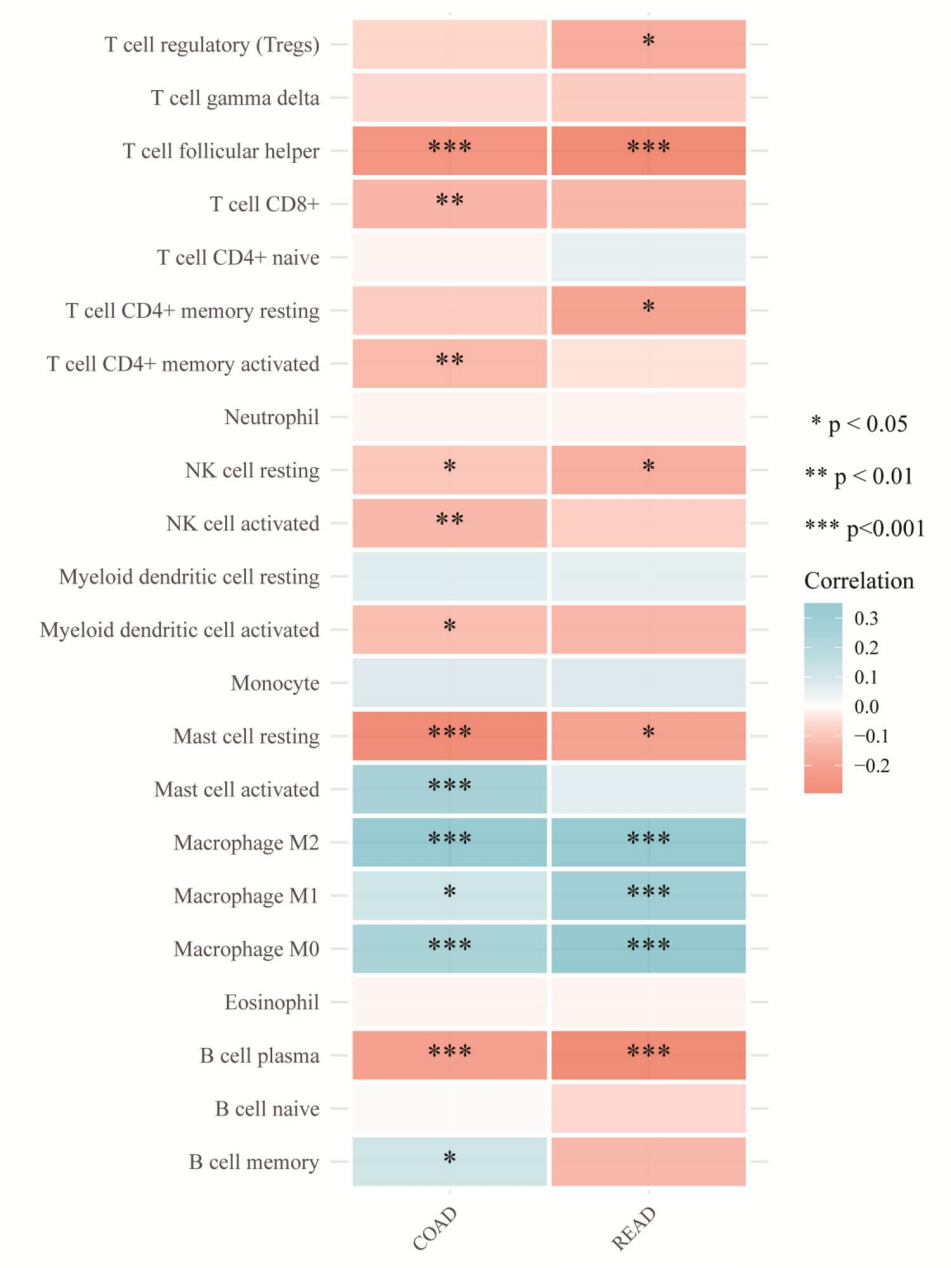
Analysis of PTGIS expression and immune infiltration. The abscissa: represents tumor tissue, the ordinate: represents different immune infiltration scores, different colors represent correlation coefficients, negative values represent negative correlations, and positive values represent positive correlations. The stronger the correlation, the deeper the color, and the asterisk represents significance (*p).

The results showed that PTGIS gene was positively correlated with Mast cell activated, Macrophage M2 and Macrophage M0 in COAD, and negatively correlated with T cell follicular helper, T cell CD8+, T cell CD4+ memory activated, NK cell activated, Mast cell resting and B cell plasma cells. In READ, it was positively correlated with M2 macrophages and M0 macrophages. In READ, it was positively correlated with M2 macrophages and M0 macrophages (Fig. 7).

### The functions of PTGIS related proteins and the biological processes involved

**Figure 8:**
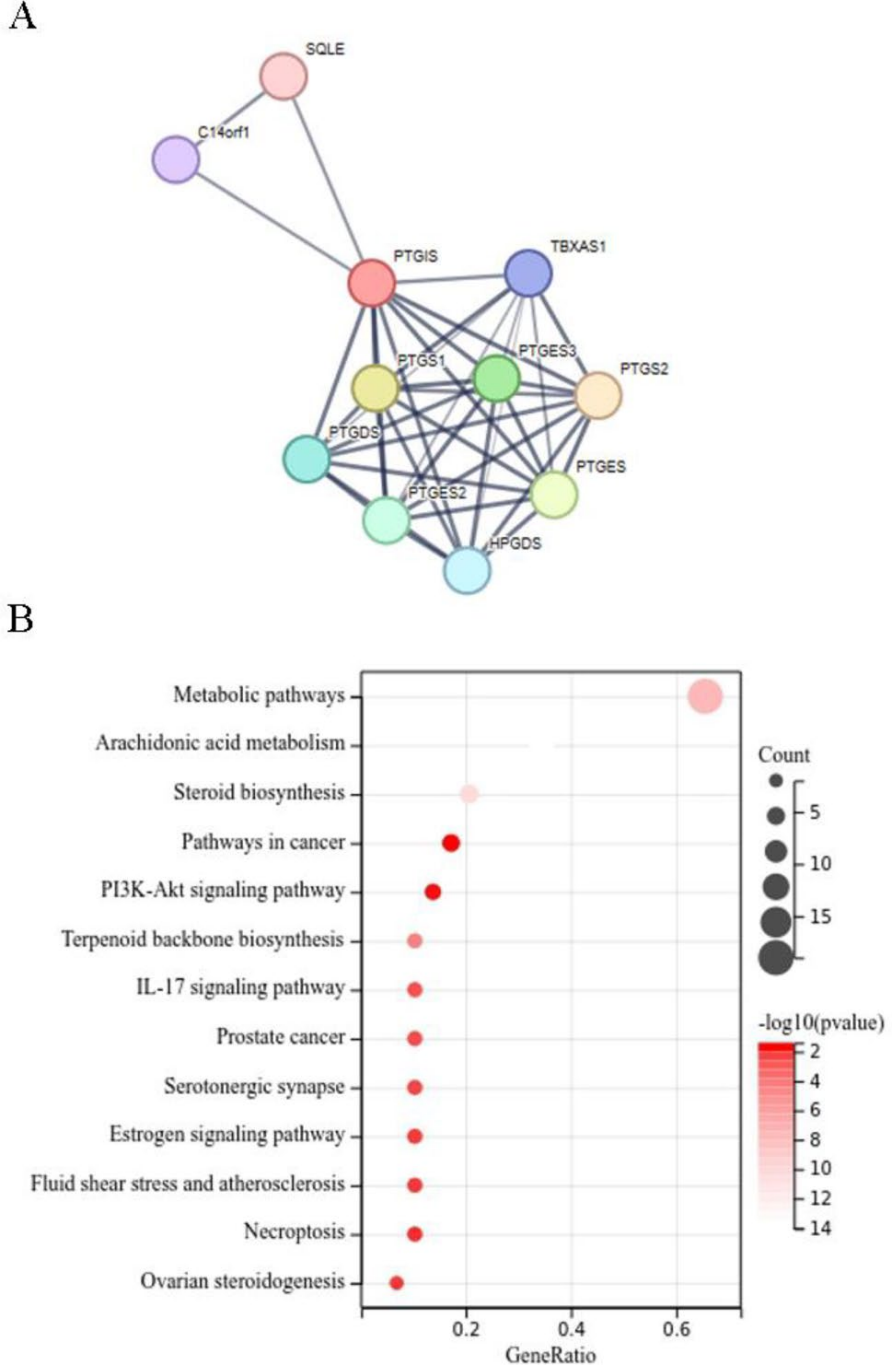
PPI networks and functional enrichment analyses. (**A**) A network of LIMK1 and its co-expression genes; (**B**) functional enrichment analyses of 31 involved genes.

In order to construct PPI networks and functional annotations, we ran a STRING database (Fig. 8A), and the KEGG results showed that a network of PTGIS pathways, mainly involved in metabolic pathways, arachidonic acid metabolism, steroid biosynthesis, cancer pathways, etc. (Fig. 8B).

## Discussion

Prostacyclin synthase (PTGIS), also known as CYP8, PGIS, PTGI and CYP8A1, is a member of the cytochrome P450 superfamily. It can catalyze the production of arachidonic acid to PGI2 and be involved in various physiological and pathological processes such as adenylate cyclase activation, cAMP elevation and protein kinase A activation, thereby playing the role of vasodilation, platelet aggregation and proliferation vascular smooth muscle cells and inhibits migration, etc^10,11^. It can regulate many physiological functions of the cardiovascular system, such as angiogenesis. Studies have shown that differential expression of PTGIS-encoding genes in many diseases is closely related to the occurrence and development of diseases such as pulmonary hypertension (PAH), hepatic fibrosis, etc. As far as we know, there have not been many studies on the functional relationship between PTGIS and tumors. Therefore, whether PTGIS plays an important role in tumors remains to be fully elucidated. Based on the online bioinformatics database, this study comprehensively demonstrated the PTGIS gene expression profiles of 31 different tumors, including gene expression and survival analysis. These results showed that PTGIS expression was significantly downregulated in 17 cancer types, ACC, BLCA, BRCA, CESC, COAD, ESCA, KICH, KIRP, LIHC, LUAD, LUSC, OV, PRAD, READ, THCA, UCEC, UCS. Among the above cancers, only five cancers prognosis were significantly correlated with the expression of PTGIS. Analysis of the survival prognosis plots revealed that high expression of ACC, BLCA, COAD, KIRP, LUSC, and PTGIS was significantly correlated with poor prognosis. It is precisely because of the discovery that the high expression of PTGIS gene is associated with poor prognosis of patients, in this study, we used colorectal cancer cells with significantly lower expression of PTGIS gene than normal colorectal tissue as a cell model to explore its pathophysiological processes of occurrence and development by overexpressing PTGIS gene. It is reported that mutations play an important role in the formation and development of tumors and are considered important targets for tumor diagnosis and treatment^12^. Through the analysis of this study, we found that PTGIS mutations are also abundant in different tumors. In particular, the mutation rate of colorectal cancer patients is the highest at over 8%. Among them, PTGIS has the highest frequency of gain type changes. Therefore, this study focuses on the relationship between PTGIS and colorectal cancer.

Currently, colorectal cancer has become a common cancer with the highest incidence and mortality in the world, and the onset is becoming younger in middle age^13^. Early treatment of colorectal cancer patients is mainly limited to surgery, radiotherapy and chemotherapy with obvious side effects and low quality of life after surgery. In recent years, many targeted drugs have emerged for the treatment of advanced colorectal cancer, including antibodies that inhibit EGFR receptor activity, such as cetuximab, panitumumab, kinase inhibitors: regorafenib, etc. However, since cancer, including colorectal cancer, is often a case Due to this very heterogeneous disease, these targeted therapies must inevitably be confronted with the problem of group benefit selection. Therefore, it is very important to further research the mechanism of occurrence and development of colorectal cancer and explore more comprehensive, accurate and effective early detection and treatment methods. Most of the factors that lead to the occurrence of colorectal cancer can be modified and the occurrence monitored, so the prediction of colorectal cancer is of great importance^14^.

In this work, we used a bioinformatics database to analyze the correlation between the PTGIS gene and the occurrence and development of colorectal cancer. It was found that the expression of the PTGIS gene was different in normal colorectal tissues and colorectal cancer tissues. We found that expression of the PTGIS gene in colorectal cancer tissue was lower than that in normal colorectal cancer tissue. The q-PCR and WB results confirmed the above results. The expression of the PTGIS gene has an impact on the prognosis of colorectal cancer, the overall survival rate of patients with low expression of the PTGIS gene was higher than that of patients with high expression of the PTGIS gene, the analysis results of the linkedOmics database match those of the GEPIA2 database. In this study, while observing the relationship between PTGIS and the proliferation and invasion of colorectal cancer cells, we found that PTGIS was normally weakly expressed in cancer cells, and SW480 cells overexpressing PTGIS had stronger proliferative ability and higher activity, decreased apoptosis and increased Invasiveness was demonstrated compared to the control group, suggesting that PTGIS gene expression differs from common tumor genes and low PTGIS gene expression leads to better prognosis. The mechanism of the PTGIS gene involved in colorectal cancer progression needs further investigation.

In the last two decades, about 20% of colorectal cancer patients have already formed metastases at the time of diagnosis^15^. Some patients classified as low-risk colorectal cancer also have some chance of recurrence or metastasis after treatment^16^. The survival rate of patients with metastatic colorectal cancer is less than 15%, while the survival rate of patients with non-metastatic colorectal cancer is over 75%^17^. It can be seen that colorectal cancer metastasis is the main factor leading to death. Tumor invasion is a key step in tumor growth and metastasis, which is of great importance for tumor treatment and prognosis. In cancer, EMT is considered to be one of the main mechanisms leading to metastasis and metastatic cancer^18^. When EMT is activated, cancer cells gain the ability to invade and metastasize^19,20^. In this study, Spearman was used to analyze the correlation between a single gene and the pathway score. It has been found that PTGIS may be related to the occurrence of the EMT mechanism in colorectal cancer. It provides ideas for further research on PTGIS and colorectal cancer.

From the results of the immune infiltration, we can find that the PTGIS gene is positively correlated with mast cell-activated macrophages M2 and macrophages M0 in COAD, which can promote the development of a tumor. In addition, it was negatively correlated with follicular helper T cells, CD8+ T cells, memory-activated T cells CD4+, activated NK cells, resting mast cells, B-cells, plasma cells that have the function of tumor killing or to inhibit. Therefore, it shows that the PTGIS gene can promote the occurrence of cancer in COAD. Furthermore, the PTGIS gene in READ was positively correlated with macrophage M2 and macrophage M0, which could promote tumor development. In addition, it negatively correlated with follicular helper T cells, CD8+ T cells, resting T cell CD4+, resting NK cells, and B cell plasma cells that had the function of inhibiting tumors. Therefore, this shows that the PTGIS gene can promote the occurrence of cancer in READ. In this study, PTGIS was positively correlated with M2 macrophages, which may indicate that PTGIS may be activated in macrophage polarization, and the expression of PTGIS will affect the activation of T cells, which is negatively correlated, indicating that PTGIS inhibits T cell function in colorectal cancer.

Finally, the associated protein network diagram and major biological processes involved in PTGIS gene were retrieved from STRING website, which confirmed that the major enriched biological processes including metabolic pathways, arachidonic acid (AA) metabolism, steroid biosynthesis, pathways in cancer etc^21,22^. The mechanism of the PTGIS gene involved in the progression of colorectal cancer needs further. PGI2 is the main metabolite of AA, and PGI2 can activate PPAR to promote the occurrence of cancer^23^. PTGIS is a key factor in the increase of PCI2 in tumors, and may affect the release of inflammatory factors through the synergistic effect of AA pathway, thus affecting the recruitment of immune cells. The above results indicate that PTGIS may play an immunosuppressive role by affecting tumor immune infiltrating cells.

However, there are some limitations in this study. We used bioinformatics methods to analyze the differences of PTGIS expression between tumor tissues and normal tissues and their effects on tumor biological characteristics, and predicted the possible downstream mechanisms of PTGIS and the effect on immune infiltration, at the same time, the in vitro experiments of cell biological function were done. Nevertheless, in order to investigate the role of PTGIS genes in the development of colorectal cancer and detailed mechanisms by which PTGIS influences immune infiltration, we need to further research the molecular mechanisms of PTGIS in colorectal cancer in vitro, and, all above results should also be verified in vivo and clinical trials (Supplementary file).

In conclusion, the PTGIS gene may play an important role in the development, invasive and prognosis of colorectal cancer, and may also play a special role in immune infiltration. At present, there are few studies on the role of the PTGIS gene in the pathogenesis of colorectal cancer. Further study on the relationship between PTGIS Gene and colorectal cancer is of great significance for finding highly specific markers for early detection of colorectal cancer and providing new targets for subsequent treatment of colorectal cancer.

### Supplementary Information

Supplementary Information.

## Data Availability

The datasets during the current study are available from the corresponding author on reasonable request.

## Abbreviations

ACC: Adrenocortical carcinoma
ANOVA: Analysis of variance
BLCA: Bladder urothelial carcinoma
BRCA: Breast invasive carcinoma
CESC: Cervical and endocervical cancers
CNA: Copy number change
COAD: Colon adenocarcinoma
EdU: 5-Ethynyl-2′-deoxyuridine
EMT: Epithelial-Mesenchymal Transition
ESCA: Esophageal carcinoma
FBS: Etal bovine serum
GAPDH: Glyceraldehyde-3-hosphate dehydrogenase
GTE_X_: Genotype-Tissue Expression
GSVA: Gene set variation analysis
IgG: Immunoglobulin G
KEGG: Kyoto Encyclopedia of Genes and Genomes
KICH: Kidney Chromophobe
KIRP: Kidney renal papillary cell
LIHC: Liver hepatocellular carcinoma
LUAD: Lung adenocarcinoma
LUSC: Lung squamous cell carcinoma
MTT: 3-(4,5)-Dimethylthiahiazo (-z-y1)-3,5-di- phenytetrazoliumromide
OS: Overall survival
OV: Ovarian serous cystadenocarcinoma
PAH: Pulmonary hypertension
PPI: Protein–Protein Interaction Networks
PRAD: Prostate adenocarcinoma
PTGIS: Prostaglandin I2 synthase
READ: Rectum adenocarcinoma
RPM: Revolutions Per Minute
RPMI: Roswell park memorial institute
RT-qPCR: Quantitative real-time polymerase chain reaction
SD: Standard deviation
THCA: Thyroid carcinoma
TPM: Transcripts Per Million
UCEC: Uterine Corpus Endometrial Carcinoma
UCS: Uterine Carcinosarcoma
WB: Western blot
