# Supplementary figures and images for: Validating the role of PTGIS gene in colorectal cancer by bioinformatics analysis and in vitro experiments
Source: Sci Rep. 2023 Oct 1;13:16496. doi: 10.1038/s41598-023-43289-2 (PMC10543560; doi:10.1038/s41598-023-43289-2)

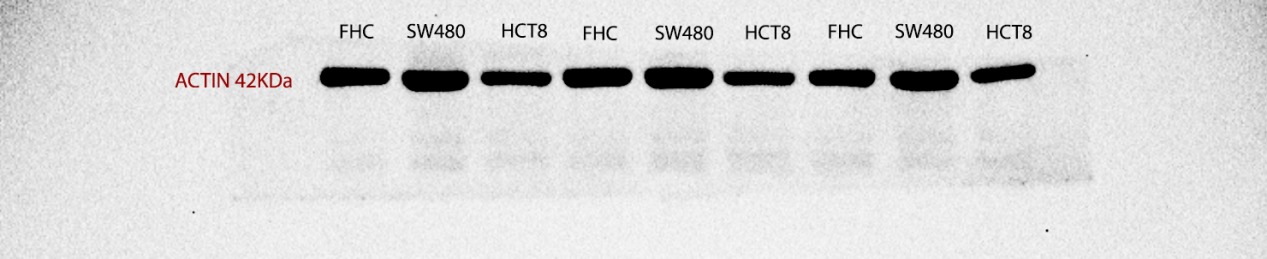


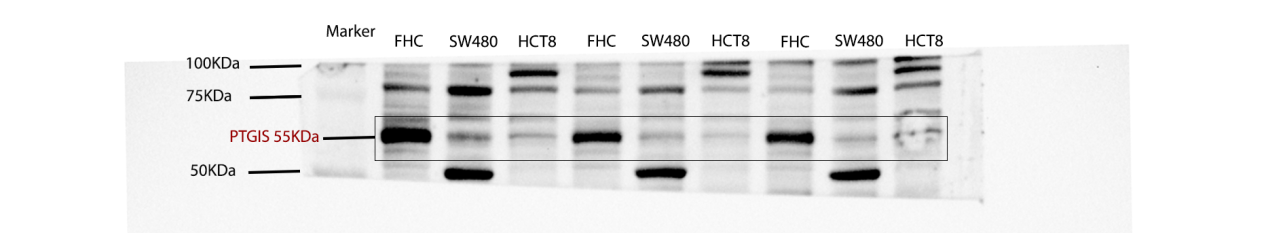

Supplement: Supplementary file 1 — Supplementary Information. [file 41598_2023_43289_MOESM1_ESM.docx]
